# Population-based risk factors and urogenital comorbidities associated with genital herpes: A nationwide study of 4 million women

**DOI:** 10.1016/j.ijregi.2024.100457

**Published:** 2024-09-21

**Authors:** Christoffer Sundqvist, Xinjun Li, Christer Borgfeldt, Per-Ola Forsberg, Kristina Sundquist, Filip Jansåker

**Affiliations:** 1 Center for Primary Health Care Research, Department of Clinical Sciences Malmö, Lund University, Lund, Sweden; b Department of Obstetrics and Gynaecology, Skåne University Hospital, Department of Clinical Science Lund, Lund University, Lund, Sweden; c University Clinic Primary Care Skåne, Region Skåne, Malmö, Sweden; 4 Center for Community-based Healthcare Research and Education (CoHRE), Department of Functional Pathology, School of Medicine, Shimane University, Matsue, Japan; 5 Department of Clinical Microbiology, Center of Diagnostic Investigations, Rigshospitalet, Copenhagen University Hospital, Copenhagen, Denmark

**Keywords:** Cervical neoplasia, Genital herpes, Herpes simplex, Sociodemographic factors, Urogenital infections

## Abstract

**Objectives:**

To explore the population-based risk factors for genital herpes in women and examine whether genital herpes occurs at higher rates in women diagnosed with cervical neoplasia and common urogenital infections.

**Methods:**

An open cohort study consisting of 4,097,075 women ≥15 years of age in Sweden (2002-2018). The outcome was genital herpes diagnosis. The predictor variables were sociodemographic factors (age, educational level, family income, region of residence, and country of origin) and urogenital comorbidities (cervical carcinoma neoplasia, cystitis, vaginosis, and vulvovaginitis) and parity. National registers and primary health care data were used. Cox regression models were used to estimate hazard ratios with 95% confidence intervals. Interactions tests were conducted.

**Results:**

A total of 15,727 women received a genital herpes diagnosis in inpatient and outpatient specialist care settings during the study period. Sociodemographic factors and parity were associated with genital herpes. than women without these comorbidities.

**Conclusions:**

Genital herpes occurs with higher rates in women of high family income, young age, Swedish origin, co-occurrence of urogenital comorbidities, and nulliparity. The findings can be used by clinicians when encountering women with these risk factors.

## Highlights

• The risk factors and comorbidities were examined in relation to genital herpes in women.
• Sociodemographic factors and parity were associated with herpes.
• Cervical neoplasia, cystitis, vaginosis, and vaginitis were associated with herpes.

## Graphical abstract

**Figure:**
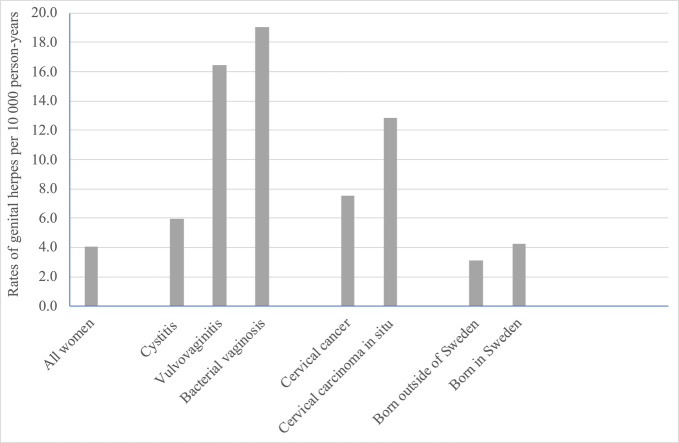

In this nationwide cohort study, sociodemographic factors, parity, and urogenital comorbidities (cervical neoplasia and common urogenital infections) were associated with genital herpes in women. For example, women born in Sweden had higher rates of genital herpes than women born outside Sweden, and women with urogenital comorbidities had higher rates than those without urogenital comorbidities.

## Introduction

Genital herpes is a prevalent sexually transmitted disease worldwide, associated with high morbidity and a large economic burden [[1], [2], [3], [4]]. It is caused by herpes simplex viruses (HSVs), primarily by HSV type 2 (HSV-2) but increasingly also by HSV type 1 (HSV-1) [3,[5], [6], [7]]. Although HSV-2 remains the most common cause of genital herpes (particularly, recurrent genital herpes), HSV-1 is becoming a leading cause of newly diagnosed genital herpes in Europe [5,6]. In Sweden, it has been estimated that every other first episode of genital herpes is caused by HSV-1 [8]. The severity of genital herpes may vary and could include very painful ulcers and (in primary infections) malaise and fever [1,2]. However, most people (up to 80-90%) infected with genital HSV are asymptomatic and unaware of the infection [1,7,9], and will, therefore, not receive a diagnosis of genital herpes [2].

The prevalence of genital herpes has been reported to be particularly high in women and varies across sociodemographic groups [3,7]. However, as recently suggested [7], population-based studies are needed to identify risk factors for improved resource planning for genital herpes. Sweden is an ideal setting for conducting such studies due to its universal health care system and sociodemographic diversity in the population. In previous research, we identified unequal sociodemographic distribution of common urogenital infections (cystitis, vaginosis, vulvovaginitis) and cervical neoplasia (cervical cancer and carcinoma *in situ*) in Sweden [[10], [11], [12]]. Possible variations in lifestyle- and behavior-related risk factors [3,7,[13], [14], [15], [16]] among sociodemographic sub-groups make it plausible that similar population-based risk factors exist for genital herpes as well. Moreover, these urogenital infections and cervical neoplasia share risk factors with genital herpes, including the mode of transmission (for HSV and human papillomavirus [HPV])—sexual contact [3,7,[13], [14], [15]]. In addition, genital herpes is also considered to be a cofactor for HPV infections in cervical carcinoma *in situ* and cancer pathogenesis [17].

All considered, it is possible that urogenital infections and cervical neoplasia are associated with genital herpes and that these associations might vary across sociodemographic groups. Considering the high prevalence [[5], [6], [7]], morbidity [[1], [2], [3], [4]], and economic burden [4] of genital herpes, identifying population-based risk factors and urogenital comorbidities for genital herpes and their potential interactions can have important clinical, public health, and research implications. Increased knowledge of risk factors and comorbidities can help clinicians who are encountering women with urogenital diseases and aid policymakers in resource allocations. Identifying interactions can increase the understanding of the interplay between behavioral risk factors and inherent biological susceptibility (e.g. sociodemographic variation in the vaginal microbiome [18]) and be used in research on potential mechanisms in these relationships.

To the best of our knowledge, no study has yet comprehensively explored the co-occurrence of urogenital comorbidities and genital herpes in a large population. This is likely due to the previous lack of longitudinal nationwide data from primary health care settings, where most urogenital infections are diagnosed. It also remains unknown whether genital herpes and other urogenital conditions could co-occur with higher rates in certain sociodemographic sub-groups of women. Using national registers and nationwide primary health care data, we aimed to explore population-based risk factors and comorbidities and their interactions with genital herpes in women.

## Material and methods

### Design, study population, and setting

This study was an open nationwide cohort study of 4,097,075 women ≥15 years of age (2002-2018) and was conducted at Lund University, Sweden. The STrengthening the Reporting of OBservational studies in Epidemiology (STROBE) checklist for cohort studies was used.

### Data sources

Data used in this study were retrieved from nationwide, comprehensive registers of high completeness, which together contain medical and sociodemographic information on the whole Swedish population. The National Patient Register [19] (NPR) was used to identify the outcome variable. NPR has been validated for use in population-based research and includes outpatient data and inpatient data of very high completeness [19]. The Total Population Register [20] was used to collect sociodemographic factors, in addition to emigration and deaths. The Swedish Medical Birth Register [21] was used to collect information on parity and the Swedish Cancer Register [22] was used to collect data on cervical neoplasia (cervical cancer and cervical carcinoma *in situ*). The Swedish Cancer Register has more than 96% completeness of cancer diagnoses, which are morphologically verified in more than 99% of cases [22]. Almost nationwide Swedish data from primary health care settings were used to identify the common urogenital infections. The coverage of the nationwide primary health care data used varied over time and region but included approximately 95% of the Swedish administrative regions in the final years of the study period [[10], [11], [12]]. All linkages between the data sources were performed using a pseudonymized version of the unique 10-digit personal identification number [23] that is assigned to each person for their lifetime upon birth or immigration to Sweden.

### Ascertainment of the outcome

The outcome was time to the first diagnosis (event) of genital herpes during the study period, defined as the 10^th^ revision of the International Classification of Diseases (ICD-10) code A600 (herpes viral infection of urogenital system, unspecified).

### Ascertainment of the predictor variables

Individual-level sociodemographic factors were collected at baseline from the Total Population Register and include the following variables: age, which was used as a categorical variable (i.e. 15-24, 25-34, 35-44, 45-64, and ≥65 years of age), educational level, which was used as a categorical variable (i.e. ≤12 or >12 years of formal education), family income, which was categorized into three groups (low, i.e. the lowest quartile, middle, i.e. the two intermediate quartiles, and high, i.e. the highest quartile) based on the annual family income divided by the number of people in the family, i.e. individual family income per capita, region of residence, which was based on three regions large cities (i.e. the six largest cities in Sweden) or in other areas in southern or northern Sweden, and country of origin, which was categorized into individuals born in or outside of Sweden (proxy for first-generation immigrant women).

Urogenital comorbidities were defined as cystitis, vulvovaginitis, and vaginosis (together called common urogenital infections in this report), as well as cervical cancer and carcinoma *in situ* (together called cervical neoplasia in this report). The urogenital infections were identified as in previous studies [24], i.e. as the first diagnosis (ICD-10 code) during the study period of each infection in almost nationwide primary health care data or NPR (outpatient data) during the study period. The ICD-10 code B373 (candidiasis of vulva and vagina), N30 (cystitis), and N768 (other specified inflammation of vagina and vulva) were used to measure candida vaginitis (referred to as vulvovaginitis), acute infective cystitis (referred to as cystitis), and bacterial vaginosis (referred to as vaginosis). The ICD-10 codes N301-4 and N308 were not used to define cystitis because these diagnoses do not include acute infective lower urinary tract infection (cystitis). There is no specific ICD-10 code in Sweden for vaginosis; however, N768 is the only mentioned code for vaginosis in the two most used clinical practice online tools in Sweden (in Swedish: internetmedicin.se and medibas.se) [25,26]. The ICD-10 diagnostic code N768 was thus used as a proxy for the medically attended condition of vaginosis. Cervical neoplasia was identified from the Swedish Cancer Register by 7^th^ revision of the International Classification of Diseases (ICD-7) codes during the study period. Occurrence of (invasive) cervical cancer was measured as ICD-7 code 171, labeled as tumor indication of malignant neoplasia of cervix uteri. Occurrence of non-invasive neoplasia of cervix uteri (in this report: cervical carcinoma *in situ)* measured as the ICD-7 code 171, labeled as tumor indication of benign neoplasia of cervix uteri. Parity (number of childbirths) was used as a covariate and collected from the Swedish Medical Birth Register and used as a continuous variable.

### Missing values

The analyses did not exclude observations with missing values (3.9%). For age and country of origin, 0.0% values were missing. Values missing for family income (3.5%), education (2.5%), and region of residency (2.0%) were included in the group with the lowest family income level, lowest education level, and living in large cities, respectively.

### Statistics

Descriptive statistics and incidence rates (per 10,000 person-years) were estimated for each predictor variable (Table 1). The incidence rates are here defined as the first registration during the study period. The study period started on January 1, 2002. Baseline occurred when a woman was 15 years of age (or immigrated). Each woman could only be included once. Person‐years were calculated until outcome (genital herpes) event, death, emigration, or end of the study period (December 31, 2018). Based on the incidence rates for each variable, Cox regression models were used to estimate hazard ratios and 95% confidence intervals. Four models were analyzed (Table 2): model 1 (univariate model), model 2 (age-adjusted model), model 3 (sociodemographic model), and model 4 (full model, including all variables). We used stepwise adjustments to examine how the different variables affected the associations between the predictors and genital herpes. Age is an important confounder based on previous research [3,7,10,12,24] and was, therefore, included in the first adjusted model (model 2). Given that urogenital comorbidities share the same risk factors as genital herpes [3,7,[13], [14], [15]], we included these comorbidities in the adjustments in the final model to examine whether urogenital comorbidities had an additional effect on the associations compared with the previous models. We also evaluated the interaction between urogenital comorbidities and sociodemographic factors. The proportional hazard assumptions were tested by plotting the incidence rates over time and by calculating Schoenfeld (partial) residuals and these assumptions were fulfilled. *P* <0.05 was defined as statistical significance. SAS 9.4 (SAS Institute Inc.; Cary, NC, USA) was used.

**Table 1:** Study population characteristics, number of events, and rates (per 10,000 person-years) of first genital herpes diagnosis in Swedish women (2002-2018).

	Total population		Genital herpes				
	No.	%	No.	%	Rate	95% CI	
**Total population**	4,097,075		15,727		4.1	4.0	4.1
**Age at baseline** (years)							
15-24	669,341	16.3	7036	44.7	11.2	10.9	11.5
25-34	671,369	16.4	3848	24.5	6.0	5.8	6.2
35-44	630,673	15.4	2223	14.1	3.4	3.3	3.6
45-64	1,167,974	28.5	2272	14.4	1.9	1.8	2.0
≥65	957,718	23.4	348	2.2	0.5	0.4	0.5
**Educational level** (years)							
≤12	2,703,502	66.0	10,138	64.5	4.0	3.9	4.1
>12	1,393,573	34.0	5589	35.5	4.3	4.1	4.4
**Family income**							
Low	1,024,000	25.0	4456	28.3	5.1	4.9	5.2
Middle	2,047,812	50.0	7912	50.3	3.9	3.8	4.0
High	1,025,263	25.0	3359	21.4	3.6	3.4	3.7
**Region of residence**							
Large cities	2,262,090	55.2	11,486	73.0	4.8	4.7	4.9
Southern Sweden	1,258,975	30.7	3172	20.2	3.2	3.1	3.3
Northern Sweden	576,010	14.1	1069	6.8	2.2	2.0	2.3
**Country of origin**							
Born in Sweden	3,264,827	79.7	13,688	87.0	4.3	4.2	4.3
Born outside Sweden	832,248	20.3	2039	13.0	3.1	3.0	3.2
**Cervical neoplasia**							
Cervical carcinoma *in situ*	75,154	1.8	979	6.2	12.8	12.0	13.6
Cervical cancer	7303	0.2	52	0.3	7.6	5.5	9.6
**Urogenital infections**							
Cystitis	1,185,462	28.9	7676	48.8	5.9	5.8	6.1
Vulvovaginitis	165,220	4.0	2938	18.7	16.4	15.9	17.0
Vaginosis	120,560	2.9	2431	15.5	19.0	18.3	19.8
**Parity**	1,877,339	45.8	7662	48.7	3.8	3.7	3.9

CI, confidence interval; No., number of events; Rate, incidence rate per 10,000 person-years.

Genital herpes events and rates were measured as the first diagnosis during the study period.

**Table 2:** The association between sociodemographic variables, urogenital conditions, parity, and first diagnosis of genital herpes (15,727 cases) in Swedish women (N = 4,097,075), 2002-2018.

Covariates	Model 1				Model 2				Model 3				Model 4			
	HR	95% CI		*P*-value	HR	95% CI		*P*-value	HR	95% CI		*P*-value	HR	95% CI		*P*-value
**Age** (ref. age ≥65 years; HR = 1)																
15-24	26.49	23.79	29.50	<.0001	26.49	23.79	29.50	<.0001	27.57	24.75	30.72	<.0001	22.26	19.97	24.82	<.0001
25-34	14.52	13.02	16.21	<.0001	14.52	13.02	16.21	<.0001	14.59	13.06	16.29	<.0001	13.89	12.40	15.54	<.0001
35-44	8.49	7.58	9.51	<.0001	8.49	7.58	9.51	<.0001	8.61	7.69	9.65	<.0001	9.30	8.28	10.45	<.0001
45-64	4.60	4.11	5.15	<.0001	4.60	4.11	5.15	<.0001	4.54	4.05	5.08	<.0001	5.03	4.49	5.65	<.0001
**Educational level** (ref. >12 years; HR = 1)	0.93	0.90	0.96	<.0001	1.04	1.00	1.07	0.0324	1.06	1.03	1.10	0.0006	1.01	0.97	1.04	0.6524
**Family income** (ref. high; HR = 1)																
Low	1.42	1.36	1.48	<.0001	0.74	0.71	0.78	<.0001	0.83	0.79	0.87	<.0001	0.82	0.78	0.86	<.0001
Middle	1.13	1.09	1.18	<.0001	0.93	0.89	0.97	0.0003	0.95	0.92	1.00	0.0265	0.95	0.91	0.99	0.0100
**Region of residence** (ref. large cities; HR = 1)																
Southern Sweden	0.56	0.54	0.59	<.0001	0.59	0.57	0.62	<.0001	0.57	0.55	0.59	<.0001	0.64	0.62	0.67	<.0001
Northern Sweden	0.40	0.37	0.42	<.0001	0.44	0.41	0.47	<.0001	0.41	0.39	0.44	<.0001	0.47	0.44	0.50	<.0001
**Country of origin** (ref. born in Sweden; HR = 1)	0.68	0.65	0.71	<.0001	0.60	0.57	0.63	<.0001	0.59	0.56	0.62	<.0001	0.56	0.54	0.59	<.0001
**Cervical neoplasia** (ref. non; HR = 1)																
Cervical cancer	1.86	1.42	2.44	<0.001	1.96	1.50	2.58	<0.001					1.67	1.27	2.19	<0.001
Cervical carcinoma *in situ*	3.37	3.16	3.59	<0.001	1.93	1.81	2.06	<0.001					1.60	1.50	1.71	<0.001
**Urogenital infection** (ref. non; HR = 1)																
Cystitis	2.06	1.99	2.12	<.0001	2.02	1.96	2.08	<.0001					1.70	1.65	1.76	<.0001
Vulvovaginal candidiasis	5.05	4.85	5.25	<.0001	3.32	3.18	3.45	<.0001					2.56	2.45	2.67	<.0001
Bacterial vaginosis	5.66	5.42	5.91	<.0001	3.61	3.46	3.77	<.0001					2.55	2.43	2.67	<.0001
**Parity** (ref. nullipara; HR = 1)	0.93	0.92	0.95	<.0001	0.95	0.94	0.97	<.0001					0.91	0.90	0.93	<.0001

CI, confidence interval; HR, hazards ratio; ref.: reference.

Model 1: univariate model; model 2: age adjusted model; model 3: adjusted for age + sociodemographic factors; model 4: full model.

## Results

A total of 15,727 of the 4,097,075 included women were diagnosed with genital herpes during the study period, corresponding to an incidence rate (per 10,000 person-years) of 4.1 (95% confidence interval 4.0-4.1).

Table 1 includes the study population characteristics, events, and the incidence rate of genital herpes for the different sub-groups. The incidence rate of genital herpes seemed to differ between the different sub-groups. The highest incidence rates were found in those women aged 15-24 years and those with cervical carcinoma *in situ,* vulvovaginitis, and vaginosis.

Table 2 shows the hazard ratios between the predictor variables and genital herpes. In model 1, women of young age, low family income, and those diagnosed with cervical neoplasia or urogenital infections were found to have a higher risk of genital herpes than their corresponding reference group, whereas women living outside large cities and being born outside of Sweden had a lower risk of genital herpes than their corresponding reference group. In model 2, the association between urogenital infections and genital herpes and cervical carcinoma *in situ* and genital herpes were somewhat attenuated but remained significant whereas the association between family income and genital herpes changed direction (i.e. after adjusting for age, women with low family income had a lower risk of genital herpes than women with high family income). In model 3, the associations between the sociodemographic factors and genital herpes were similar to the associations in the previous age-adjusted model 2. In model 4, the associations between age and genital herpes were somewhat attenuated, as were the associations between urogenital infections and genital herpes, as well as urogenital neoplasia and genital herpes. Except for the association between education level and genital herpes, all associations remained statistically significant throughout all models.

Table 3 shows the association between cervical neoplasia, urogenital infections, and genital herpes by country of origin. The interaction tests are presented as ratios between the hazard ratios for those born in Sweden and those born outside Sweden. The association between cervical cancer and genital herpes seemed to be stronger in Swedish-born women, whereas an opposite pattern appeared among women born outside Sweden for cervical carcinoma *in situ*.

**Table 3:** The association between urogenital comorbidities and first diagnosis of genital herpes by country of origin in Swedish women, 2002-2018.

Covariates	Born in Sweden			Born outside Sweden			Ratio between Sweden/born outside Sweden		
	HR	95% CI		HR	95% CI		Ratio	95% CI	
**Cervical neoplasia** (ref. Non; HR = 1)									
Cervical cancer	1.68	1.27	2.23	1.40	0.53	3.75	1.20	0.43	3.32
Cervical carcinoma *in situ*	1.53	1.43	1.64	2.48	2.02	3.05	0.61	0.50	0.76
**Urogenital infection** (ref. non; HR = 1)									
Cystitis	1.68	1.63	1.74	1.79	1.64	1.96	0.94	0.88	1.00
Vulvovaginitis	2.52	2.41	2.64	2.83	2.54	3.15	0.89	0.81	0.98
Vaginosis	2.48	2.36	2.61	2.86	2.54	3.22	0.87	0.78	0.97

CI, confidence interval; HR, hazard ratio; ref., reference.

Fully adjusted for all covariates including sociodemographic factors and parity (see Table 2).

## Discussion

This nationwide study of 4 million women in a country with universal healthcare contributes to the understanding of population-based risk factors and urogenital comorbidities associated with genital herpes. The main findings were that several associations between the examined factors and genital herpes were observed and that some of these associations varied by country of origin. This is important new knowledge that can be used to identify risk and protective mechanisms for genital herpes. The findings in this study are valuable for clinicians who are encountering women with symptoms of urogenital infections, as well as health care planners. To the best of our knowledge, these combined approaches have previously not been applied when exploring possible risk factors and comorbidities and their interactions regarding genital herpes.

The findings that cervical neoplasia, cystitis, vaginosis, and vulvovaginitis are urogenital comorbidities associated with genital herpes were expected because these urogenital conditions share many established lifestyle risk factors, including sexual contact where cervical neoplasia is caused by HPV infection and genital herpes is caused by HSV infection [3,7,[13], [14], [15]]. The co-occurrence of genital herpes and urogenital infections may also partly be due to biological mechanisms, by which the vaginal microbiome could affect the susceptibility for urogenital infections and the progression of HPV infection to cervical neoplasia [18,[27], [28], [29], [30], [31], [32], [33]]. Thus, it is possible that an interplay between lifestyle factors, especially sexual behavior, and biological mechanisms are involved in the co-occurrence of urogenital infections observed in this study. Moreover, sociodemographic differences in lifestyle factors [3,7,[13], [14], [15], [16]] could possibly explain the association between individual-level sociodemographic factors and genital herpes. It is also possible that first-generation immigrant women are less prone to seek health care for symptoms of genital herpes compared to Swedish-born women. Although we do not have access to factors that could elucidate the underlying mechanisms behind our findings, the associations between other urogenital infections and genital herpes, which vary by country of origin, suggest a potential interaction between behavioral risk factors and possibly a biological susceptibility (e.g. vaginal microbiome). Another findings was that women born in Sweden and first-generation immigrant women had a higher risk for genital herpes when they also had urogenital comorbidities, which primarily could be explained by shared risk factors between genital herpes and the urogenital comorbidities [3,7,[13], [14], [15]]. The lower risk of genital herpes among women born outside Sweden may also be explained by differences in risk behaviors. Altogether, the association between sociodemographic factors and urogenital infections and genital herpes and their interactions posit important new insights into possible underlying mechanisms that warrants further studies.

Our findings have potential clinical and public health implications. For example, increased awareness of urogenital morbidity and variations by sociodemographic factors is important knowledge for clinicians treating women with symptoms of urogenital infections. However, although most findings are likely to be generalizable to other countries, additional research conducted in different health care systems is needed before the findings can be useful in other clinical settings. For example, considering the worldwide disproportional distribution of genital herpes [3,7], it is possible that genital herpes clusters in certain sociodemographic subpopulations worldwide, which, however, not necessarily need to be consistent within different countries. The findings in our study, which suggest that the distribution of genital herpes varies by sociodemographic factors in Sweden, adds additional knowledge to the preventive work for reducing urogenital infections in women. Although genital herpes often is self-limiting, all women with genital herpes should receive a confirmed diagnosis and proper treatment to reduce the discomfort of outbreaks and recurrences [3,7].

The main limitations of this study are that microbiological data were not available and that we were unable to measure the true occurrence of genital herpes (because register data underestimates genital herpes) [3,7]. Because we do not have access to data on symptoms nor serological or microbiological tests, it is also possible that misdiagnosis between genital herpes and other urogenital infections might have occurred to some degree. In addition, we did not have access to data on whether HSV-1 [6] or HSV-2 [5] was the cause of the outcome in our study. However, we only measured the first diagnosis of genital herpes in the women during the study period, and a previous, smaller study from Sweden showed that about half of all first episodes of genital herpes were caused by HSV-1 [8]. Moreover, although we have collected several variables that were stepwise adjusted for in the models, unmeasured confounding because of lack of behavioral risk factors might exist.

This study also has several strengths which balance the limitations. The study is a nationwide study on population-based risk factors for genital herpes, adjusted for common urogenital infections and other known risk factors. It is based on comprehensive data from validated nationwide registers [[19], [20], [21], [22]] and almost nationwide primary health care data [[10], [11], [12]]. This approach has, to the best of our knowledge, not been applied before in studies on genital herpes and limits the risk of recall bias. Because of the personal identification number [23] for each individual, there was no loss to follow-up otherwise often seen in observational studies. Moreover, the finding of young age as a risk factor for genital herpes was expected because most women worldwide are diagnosed with a first episode of genital herpes at a young age [3,7], indicating a high validity of our data.

## Conclusion

Genital herpes occurs in higher rates among women born in Sweden and those who also experience cervical neoplasia, cystitis, vaginosis, and vulvovaginitis, which may be due to shared risk factors. Altogether, our findings suggests that certain sub-groups of women disproportionally experience urogenital morbidity through adulthood in Sweden and that sociodemographic differences and differences in other risk factors might be the driving explanations behind these findings, which we believe are useful for health care planners and clinicians. Future research is warranted to determine underlying mechanisms and their interactions.

## Declarations of competing interest

The authors have no competing interests to declare.
